# Antiviral activity of Piscidin 1 against pseudorabies virus both *in vitro* and *in vivo*

**DOI:** 10.1186/s12985-019-1199-4

**Published:** 2019-07-31

**Authors:** Han Hu, Nan Guo, Shuhua Chen, Xiaozhen Guo, Xiaoli Liu, Shiyi Ye, Qingqing Chai, Yang Wang, Binlei Liu, Qigai He

**Affiliations:** 1 State Key Laboratory of Agricultural Microbiology, College of Veterinary Medicine, Huazhong Agricultural University, Wuhan, Hubei China; 2 Key Laboratory of Fermentation Engineering (Ministry of Education), National “111” Center for Cellular Regulation and Molecular Pharmaceutics, Hubei Provincial Cooperative Innovation Center of Industrial Fermentation, College of Bioengineering, Hubei University of Technology, Wuhan, 430068 China; 3 Pig health substantial innovation center, Wuhan, Hubei China; 4 Feinberg school of medicine, northwestern university, Boston, MA USA

**Keywords:** Antimicrobial peptides, Pseudorabies virus, Piscidin, Porcine epidemic diarrhea virus, *In vivo*

## Abstract

**Background:**

Swine-origin virus infection spreading widely could cause significant economic loss to porcine industry. Novel antiviral agents need to be developed to control this situation.

**Methods:**

In this study, we evaluated the activities of five broad-spectrum antimicrobial peptides (AMPs) against several important swine-origin pathogenic viruses by TCID_50_ assay. Plaque reduction assay and cell apoptosis assay were also used to test the activity of the peptides. Protection effect of piscidin against pseudorabies virus (PRV) was also examined in mouse model.

**Results:**

Piscidin (piscidin 1), caerin (caerin 1.1) and maculatin (maculatin 1.1) could inhibit PRV by direct interaction with the virus particles in a dose-dependent manner and they could also protect the cells from PRV-induced apoptosis. Among the peptides tested, piscidin showed the strongest activity against PRV. Moreover, *in vivo* assay showed that piscidin can reduce the mortality of mice infected with PRV.

**Conclusion:**

*In vitro* and *in vivo* experiments indicate that piscidin has antiviral activity against PRV.

## Background

Swine-origin virus infection is one bottleneck for the development of the porcine industry worldwide. The pathogenic viruses isolated from pigs including pseudorabies virus (PRV), porcine reproductive and respiratory syndrome virus (PRRSV), porcine epidemic diarrhea virus (PEDV), transmissible gastroenteritis virus (TGEV), and rotavirus (RV) are commonly observed in China. The viral infection is also responsible for the secondary infection by bacteria which has greatly increased the application of antibiotics [1, 2]. In the past decades, the efforts to alleviate pig viral diseases have focused on the development of vaccines to enhance the adaptive immunity of the hosts [3, 4]. However, some of the evolving viruses could escape from the host immunity through different kinds of strategies. Several viral diseases have broken out in recent years among pig herds, such as the reemergence of swine-origin influenza in 2009 [5], PED in late 2010 [6] and Pseudorabies in 2012 [7]. Thus, there is an urgent need to develop novel potential agents to kill these viruses or block their infection.

AMPs are common host defense molecules in nearly all forms of life. Ever since their discovery, AMPs have gained worldwide attention as important alternatives in the field of disease prevention and immune modulation [8, 9]. Previous studies mostly focused on the protection effect of AMPs against bacterial and fungal infection [10]. Later on, AMPs were also reported to be effective against viral infection [11]. It has been reported that some alpha-helical peptides can act as virucidal agents. For example, caerins and maculatins isolated from amphibian skin could completely inhibit human immunodeficiency virus type 1 (HIV-1) infection after virus is exposed to peptides within minutes [12]. Piscidins, discovered in the mast cells of fish, could reduce the infectivity of several important fish-origin viruses [13]. Indolicidin, a natural 13-amino acid antimicrobial peptide isolated from bovine neutrophils, could directly kill HIV-1 [14]. Bovine lactoferricin derived from bovine lactoferrin has been reported to exert antiviral activity to fight against human cytomegalovirus (HCMV), herpes simplex virus type 1 (HSV-1), HSV-2, and adenovirus [15]. Other antimicrobial agents, such as human defensins which is a group of beta-sheet peptides, can inhibit enveloped viruses such as HSV-1 and HSV-2, HIV-1, vesicular stomatitis virus (VSV), influenza virus, and HCMV by directly inactivating viruses [16]. Therefore, AMPs might be promising agents against viral infections.

PRV, a large enveloped DNA virus, is a swine neurotropic herpesvirus [17]. Although the pigs are the natural reservoir for the virus, most mammals and some avian species are also susceptible to PRV. PRV infected animals may die from central nervous system disorders [18]. PRV infection poses a severe threat to pig industry and either attenuated live or inactivated vaccines are usually used to control the disease [19]. Although vaccination can suppress development of the disease, vaccines cannot eliminate virus infection. Mutant isolates emerged and caused Pseudorabies outbreak in 2012 [7]. Thus, novel antiviral agents should be developed as a complementary to vaccination.

In one of our recently published papers, we described the antiviral activity of caerin against porcine epidemic diarrhea virus (PEDV) *in vitro* [20]. In this study, we investigated the activity of five AMPs including piscidin, caerin, maculatin, lactoferricin B, and indolicidin against several porcine-origin viruses. More inhibitory activity of piscidin, caerin, and maculatin against PRV was also explored. The *in vivo* protection effect of piscidin against PRV infection was further investigated. Chen et al found that piscidin has the anti-inflammatory and anti-nociceptive properties in inflammatory animal models [21]. Kumar et al reported the obvious anti-endotoxin and anti-bacteria properties of piscidin-1 analogues both *in vitro* and *in vivo* [22].

## Methods

### Peptides

The peptides (Table 1) used in this study were synthesized on an automated solid-phase peptide synthesizer by Neweast Biosiences Inc. (Wuhan, China) [23]. The crude peptides were purified via a reverse-phase high-pressure liquid chromatography (RP-HPLC) using a C_18_ column (Waters Xbridge). The elution was conducted using a water-acetonitrile linear gradient (0–80% of acetonitrile) containing 0.1% (V/V) trifluoroacetic aicd (TFA). Finally, the purity and accurate masses of the product peptides were determined using HPLC and mass spectrometry, respectively.

**Table 1:** Antimicrobial petides used in this study

Peptides	Sequence	No. of the amino acids	Molecule weight
Caerin	GLLSV LGSVA KHVLP HVVPV IAEHL	25	2584
Maculatin	GLFGV LAKVA AHVVP AIAEH F	21	2144.5
Lactoferricin B	FKCRR WQWRM KKLGA PSITC VRRAF	25	3124.2
Piscidin-1	FFHHI FRGIV HVGKT IHRLV TG	22	2569.3
Indolicidin	ILPWK WPWWP WRR	13	1904.4

### Viruses and cells

The PRV strains of Ea, HNXX, 152, and TGEV WH-1 were propagated in PK-15 cells based on the method reported in previous studies [24]. RV TM-a was propagated in Rhesus monkey kidney cell line (MA104) [25]. PRRSV YA was cultured in Marc-145 cells [26]. PEDV strain CH/YNKM-8/2013 was cultured in vero cells as described before [27]. The virus suspension was stored at − 80 °C until used.

### Measurement of the virucidal effect of the AMPs

In our initial topical screening assays, the viruses were pre-incubated with peptides (50 μg/ml) for 1 h at 37 °C before they were added to the target cells. Peptide-free controls (virus plus cell culture medium only) were incubated in parallel. After incubation, the TCID_50_ of peptide-treated viruses and peptide-free viruses was measured. Residual virus was calculated according to the following formula, residual infectivity = b/a × 100% (“a” represent TCID50 of residual virus non-treated by the AMPs (control), “b” represent TCID50 of residual virus after treated by AMPs).

### Cytotoxicity assay

A 3-(4, 5-dimethylthiazol-2-yl)-2, 5-diphenyltetrazolium bromide (MTT) cell proliferation assay was used to assess cell viability as described previously [28]. Briefly, PK-15 cells were added to 96-well tissue-culture plates (Coster, USA) and incubated overnight at 37 °C. Serial two fold dilutions of AMPs ranging from 200 to 3.12 μg/ml were added into the plate. The cells were incubated with AMPs at 37 °C for 48 h, and the morphology of the cells was evaluated under light microscope. Then, the cells were further incubated with MTT solution for 4 h and the cell pellets were collected for measurement of absorbance at 490 nm by an ELISA reader.

### Plaque reduction assay

The antiviral activity was evaluated by plaque reduction assay [29] at different infection stages: pre-infection and post-infection, as well as direct inactivation of the viruses. Briefly, the direct effect of peptides on virus per se was analyzed by the following procedures. Monolayer of PK-15 was prepared in 12-well tissue culture plates. Prior to infection, 300 μl of different virus stocks with the titer of 10^3^ pfu/ml were incubated with 33 μl of peptides diluted in DMEM at concentrations of 25, 5, 1, 0.2 μg/ml at 37 °C for 1 h. After incubation, the peptide-virus mixture was further diluted in DMEM and was inoculated to the monolayer of PK-15. The absorption proceeded at 37 °C for 1 h. The virus-peptide mixture was then removed and replaced with the overlaid medium (4% FBS and 1.5% carboxymethyl cellulose in DMEM). The plaque formation was measured on the 3-4th days later by crystal violet solution staining.

To analyze the post-infection effect of the peptides on virus growth, cells were infected with the same amount of virus mentioned above at 37 °C for 1 h. Subsequently, the cells were washed 3 times with DMEM and treated by the peptides at different concentrations for 1 h at 37 °C. The peptides were removed from the cell culture and replaced with the overlaid medium. The resulting PFU titer was determined as described above.

To understand the pre-infection effect of the peptide on virus, the cells in 12-well tissue culture plates were treated with peptides at serial concentrations at 37 °C for 1 h. Then, cells were washed with DMEM for 3 times, followed by infection with viruses. The cells were covered with the overlaid medium for plaque assays.

### Cell apoptosis inhibited by the AMPs

Cell apoptosis was analyzed with Annexin V-FITC kit (Nanjing Keygen Biotech. Co., Ltd). Briefly, FITC-conjugated Annexin V (50 μl/well) and propidium iodide (PI, 50 μl/well) were added to the cells infected by AMP-treated viruses. Then, the obtained mixture was incubated at room temperature for 15 min in the dark prior to fluorescence observation. Cell apoptosis was determined on basis of the observation soon after initiating apoptosis. Cells translocated the membrane phosphatidylserine (PS) from the inner surface of the plasma membrane to the cell surface. Thereafter, PS can be easily detected by staining with a fluorescent conjugate of Annexin V (green), a protein that has a high affinity to PS. Cellular late apoptosis was determined by cell staining with PI (red). The results were analyzed by fluorescence microscope (Nikon, Japan) at 36 hpi.

### *In vivo* PRV challenge assay

The 6 to 8 week-old specific-pathogen-free BALB/c mice were purchased from the Experimental Animal Center of Zhongnan Hospital of Wuhan University (China) and randomly divided into six groups consisting of 10 mice each. Individuals in each group of mice were anesthetized with 1–3% isoflurane gas and challenged by the intra-footpad injection with 50 μl of DMEM containing 5 × 10^3^ TCID_50_ of PRV-Ea in the presence or absence of 10, 5, 1, 0.2 μg/ml piscidin. After 14 days, the surviving mice were challenged with 5 × 10^3^ TCID_50_ of PRV again. Mice viability and behaviors were monitored on a daily basis. In the first 2 days after the challenge, the mice commonly did not exhibit severe symptoms. However, mice gradually showed neurological symptoms in the subsequent days during the monitoring period and the post-challenged mice were monitored since day 3 post PRV infection once every 6–8 h for 8 days to obtain the survival information of the mice. The level of PRV infection symptoms was scored for every mouse by the following system: 0 = posture normal, absence of neurological symptoms, 1 = mild neurological symptoms: excitation, unrest, occasional itching, and foot swollen; 2 = severe neurological symptoms: ataxia, severe pruritus, and self-mutilation, biting and bleeding of the footpad. Mice were euthanized in the chamber with CO2 gradually filled when the score reached 2.

On day 6, three mice from control group and non-control groups were euthanized and brain tissues were collected and transferred to 4% paraformaldehyde. The tissues were dehydrated in ascending grades of ethyl alcohol i.e. 70, 90 and 100% ethanol. Afterwards, the tissues were cleared with xylene. Slides were stained with Haematoxylin and Eosin (H&E) stain and analyzed through a light microscope.

All of the animal experimental protocols were approved by the Ethics Committee of Huazhong Agricultural University according to Hubei province Laboratory Animal Management Regulations (HZAUMO2015–0015). During the experiments, mice were offered ad libitum access to water and food in a controlled environment of a 12 h light/dark cycle. All efforts have been made to reduce suffering of animals.

## Result

### Antiviral activities of the peptides against swine-origin viruses

**Fig. 1:**
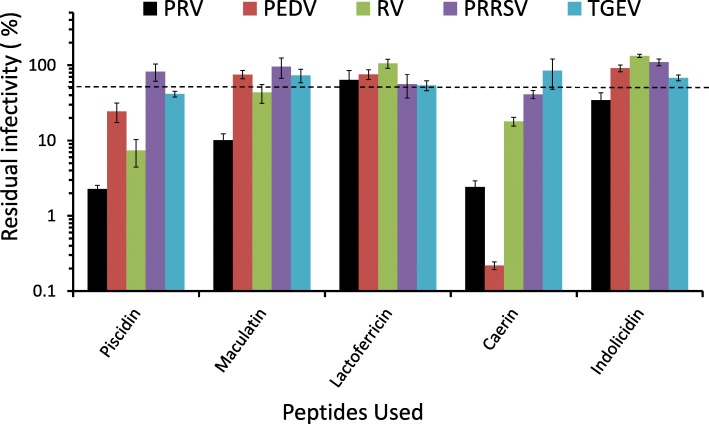
Spectrum of antiviral activity of the peptides (TCID_50_ assay). Peptides (50 μg/ml) and viruses were incubated at 37 °C for 1 h before they were added to the target cell monolayers. After incubation for 48–72 h, TCID_50_ of the virus was recorded. The bars represent ± SE. Three separate assays were conducted (*n* = 3). The dashed line means 50% residual infectivity

The antiviral effects of the peptides (maculatin, caerin, piscidin, lactoferricin B, indolicidin) were investigated *in vitro* against several viral pathogens that severely threaten the porcine industry. Four enveloped viruses were used in this assay including the PRV Ea strain, PEDV YNKM strain, TGEV WH-1 strain, and PRRSV YA isolate, as well as one non-enveloped RV TM-a strain. The peptides exhibited different inhibitory activities against these viruses (Fig. 1). The relative potency of the peptides against PRV was: piscidin≈caerin>macualtin>indolicidin> lactoferricin B. Piscidin and caerin showed strong virucidal activity with the residual infectivity being around 2.3% while indolicidin and lactoferricin B exhibited mild antiviral activity. In descending order of potency, the peptides against PEDV ranked as follows: caerin>piscidin> maculatin>lactoferricin B > indolicidin. Caerin had the most potent activity with the residual infectivity being 0.2%. The assay results indicated, that the order of potency versus PRRSV was: caerin>lactoferricin B > piscidin>maculatin>indolicidin. Caerin exhibited the best inhibitory activity with the residual infectivity being 41.1%, separately. The assay results of antivirus activity against TGEV indicated the activity order as follows: piscidin>lactoferricin B > indolicidin>maculatin>caerin. Piscidin was the most potent peptide versus TGEV with the residual infectivity being 41.5%. The assay result also indicated that PRRSV and TGEV were less sensitive to the tested peptides, compared with other viruses (Fig. 1). When it comes to RV, the relative potency order was: piscidin>caerin>maculatin>lactoferricin B > indolicidin. Piscidin displayed the most potent activity against RV with residual infectivity being 7.4%. Piscidin and caerin exhibited activity against almost all the viruses with the residual infectivity being lower than 50% with exception of piscidin versus PRRSV and caerin versus TGEV. Caerin showed the greatest inhibitory activity against PEDV with the residual infectivity being 0.2%, which was the lowest value among all the viruses tested. Maculatin exhibited strong activity against PRV (10.1%) and mild activity against RV (43.4%). However, it showed limited activity against PEDV, PRRSV, and TGEV. Lactoferricin B and indolicidin were not as potent as other peptides to fight against viruses. Lactoferricin B showed stronger inhibitory activity against TGEV than against other tested viruses with residual infectivity being 54.1%, and residual infectivity of indolicidin versus PRV was 34.6%.

### Cytotoxicity

**Fig. 2:**
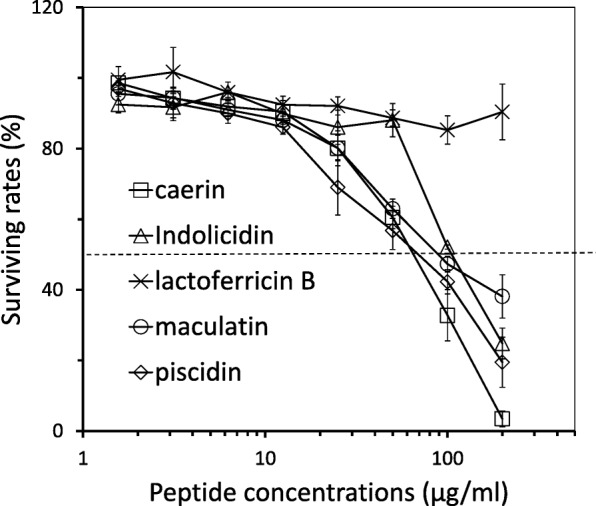
Cytotoxic properties of the peptides. PK-15 cell monolayers were incubated with peptides. The cytotoxicity was measured by MTT assay (*n* = 3). The cell survival rates at different peptide concentrations were plotted and the dashed line means 50% cell survival

We examined the effects of AMPs on cell viability after incubating PK-15 cells with different concentrations of each peptide. Cytotoxicity was evaluated using MTT method (Fig. 2). The non-linear regression analysis result indicated that the 50% toxic concentration (TC_50_) of caerin, maculatin, piscidin, and indolicidin were 67.8, 76.6, 65.2, and 110.2 μg/ml, respectively. Indolicidin was less cytotoxic than piscidin, caerin and maculatin. Lactoferricin B exhibited the weakest cytotoxic activity and it did not exhibit obvious cytotoxic activity even at maximum concentration of 200 μg/ml.

### Confirmation of inhibitory activity against PRV

**Fig. 3:**
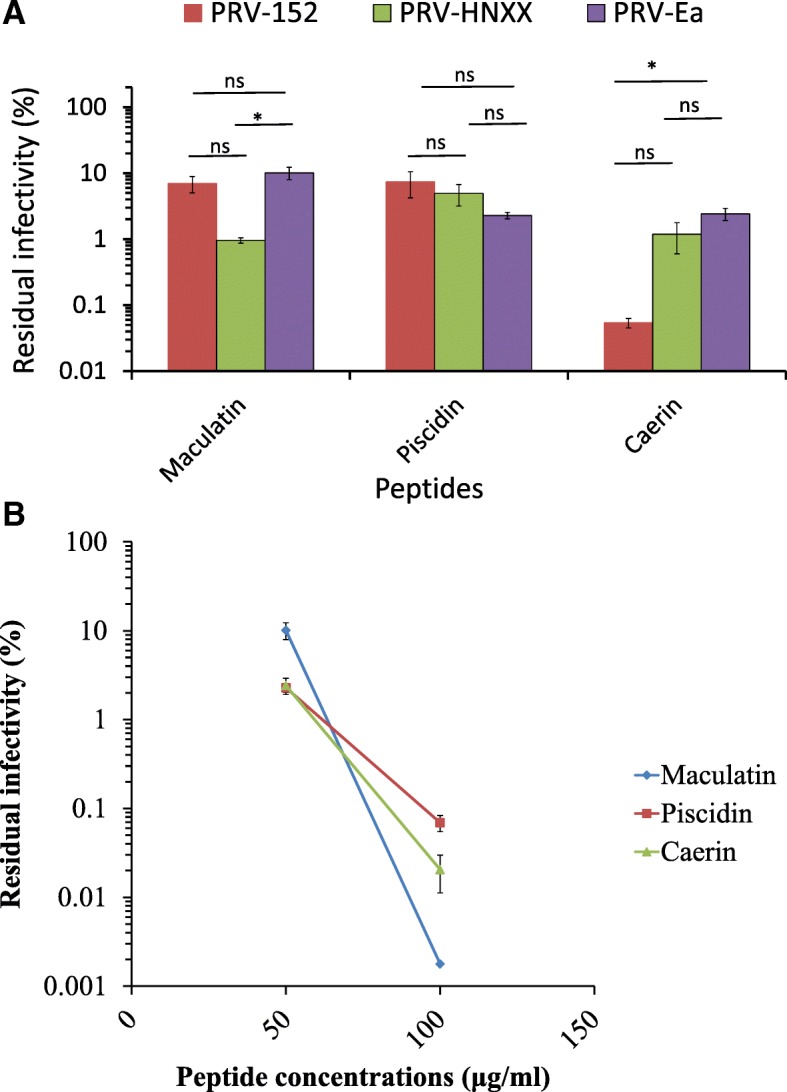
PRV inhibitory activity displayed by maculatin, piscidin and caerin (TCID_50_ assay). **a** PRV of Ea, HNXX strain and 152 isolate were treated with the peptides (50 μg/ml) for 1 h before they were added to the cell monolayers. The bars represent means ± standard errors of the means of three separate experiments (*n* = 3). *, Statistically significant difference by one-way ANOVA with Tukey post hoc test (*P* < 0.05), NS means not significant. **b** PRV Ea was treated with peptides (100 μg/ml) for 1 h before added to the cell monolayers. The effects of the peptides at 50 μg/ml and at 100 μg/ml against PRV Ea were compared

To determine whether the inhibitory activity against PRV by maculatin, caerin, piscidin was strain-specific or not, we tested the activity of these peptides against different PRV isolates including PRV-HNXX (a newly PRV isolate in China in 2012) and PRV-152 (modified Bartha strain). We found that all the three peptides displayed inhibitory activities against all the tested strains (PRV HNXX, PRV 152, and PRV-Ea) (Fig. 3a). This finding indicated that the activity of the peptides versus PRV was not strain-specific. When the concentration of the peptides increased from 50 to 100 μg/ml, the residual infectivity of PRV decreased (Fig. 3b). This trend was the most obvious for maculatin with the residual infectivity decreasing from 10.11 to 0.0017%, while the groups treated with piscidin and caerin exhibited a decrease by more than 30 folds in the residual infectivity. These data indicated that these peptides inhibited the virus in a dose-dependent manner.

### Inhibitory effect of the peptides on different stages of PRV infection

In order to better understand the inhibitory effect of AMPs on the propagation of PRV-Ea, we examined whether AMPs directly damaged virus particles or indirectly interacted with the host cells pre- or post-infection. Plaque-reduction assay was also performed to confirm the peptides’ antiviral activity.

**Fig. 4:**
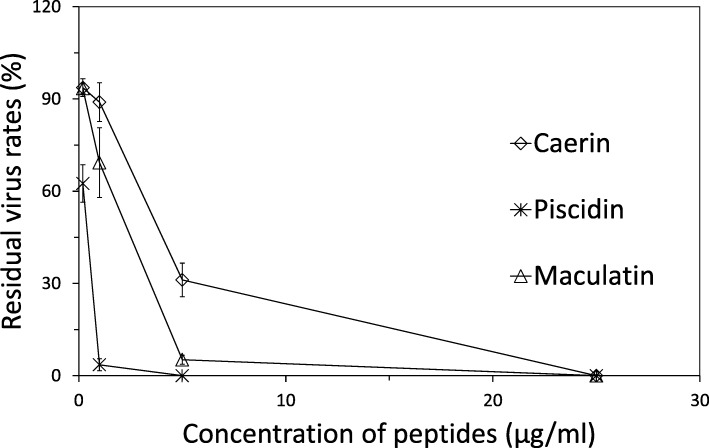
Inhibition of PRV (plaque reduction assay). Viruses were incubated with the peptides for 1 h and then added to PK-15 cell monolayers. Plaques were counted 48-72 h post incubation. Values of three separate assays are shown as means ± SEM (n = 3)

The viruses were incubated with the peptides prior to infection. The result showed that all the three peptides inhibited the virus infection in a dose-dependent manner (Fig. 4). At the concentration of 25 μg/ml, pisicidin, caerin and maculatin were found to inhibit most of the PRV particles. Pisicidin exhibited the strongest activity and obvious inhibitory ability at concentrations above 5 μg/ml. The 50% effective concentration (EC_50_) values of the peptides were calculated using probit analysis by IBM SPSS (version 21, New York, USA). The analysis revealed that EC_50_ values of caerin and maculatin were 2.63 μg/ml and 1.09 μg/ml, respectively. EC_50_ of piscidin was the lowest (0.23 μg/ml) among the peptides tested, which indicated that piscidin was the most potent antiviral peptide against PRV in this study.

Subsequently, the experiments were conducted to further determine whether the peptides could function at post- (curative) or pre-infection (prophylactic) stage. The cell monolayers were treated with piscidin, caerin, and maculatin before (prophylactic) or after (curative) the virus binding stage, separately. No inhibitory effect of the three peptides on PRV could be detected (the obtained data not shown). This implied that these peptides probably inhibited PRV infection by directly interacting with the virus particles.

### Peptides blocked cell apoptosis induced by PRV infection

**Fig. 5:**
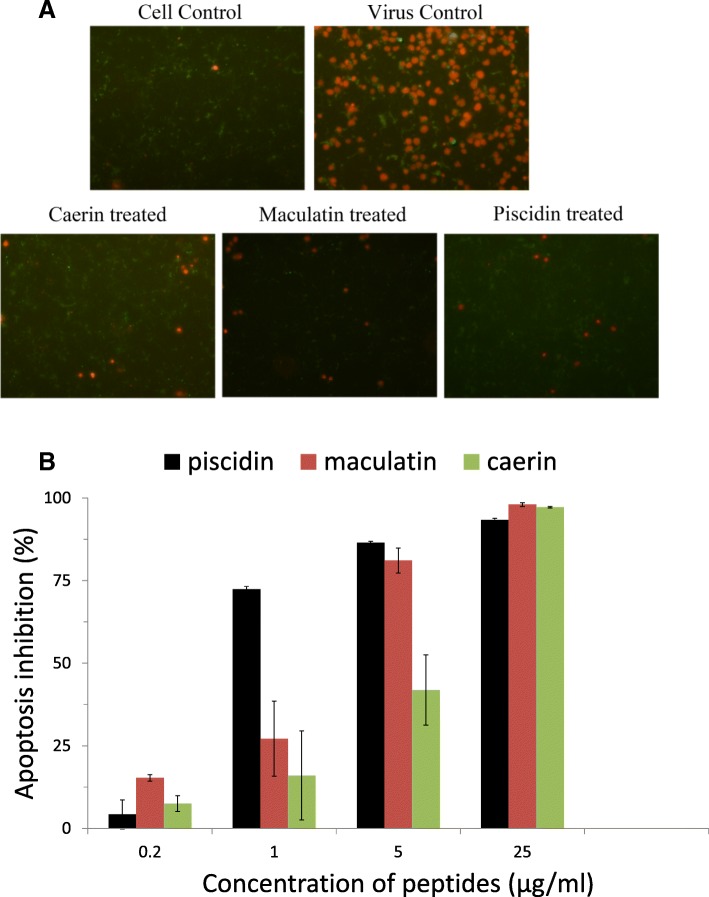
Effect of the peptides on cell apoptosis was analyzed after the cells were infected by peptide-treated viruses by FITC-conjugated Annexin V (Green) and propidium iodide (Red) staining. The red fluorescence signals are designated as the index of late apoptosis and green fluorescence signals are designated as the index of early apoptosis. The number of PI positive cells was counted to calculate the cell apoptosis inhibition rates. **a**. Staining picture displaying cell apoptosis induced by PRV treated with peptides at 25 μg/ml; **b**. Cell apoptosis inhibition rates of various groups. The bars represent means± standard errors of three separate experiments  (*n* = 3)

The cell apoptosis assay showed that PRV could induce both early and late apoptosis of PK-15 cells (Fig. 5). The late apoptosis rate of cells infected by the peptides-treated viruses decreased as the concentration of the peptide increased (Fig. 5b). The inhibition of late cellular apoptosis was obvious at the concentration of 25 μg/ml (Fig. 5a).

### Co-injection of piscidin and PRV rescued mice from PRV-Ea infection

**Fig. 6:**
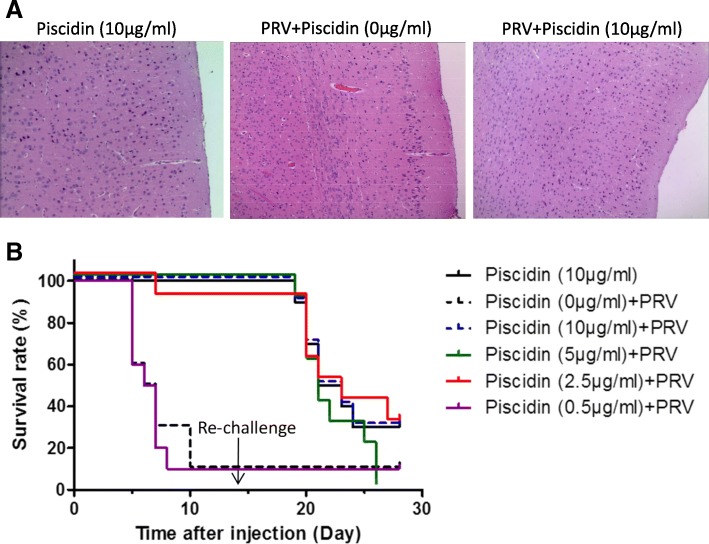
Piscidin protects mice from PRV-induced death. **a**. processed brain sections from control, co-treated, and PRV infected groups were subjected to Haematoxylin and Eosin (H&E). The representative H&E results were shown. **b**. The mice injected with piscidin or PRV, co-treated with piscidin and PRV were divided into different groups. Surviving mice were challenged with PRV on day 14 again. Survival status of control and experiment groups were monitored on a daily basis for 28 days (*n* = 10). In the group of piscidin (10 μg/ml), 2 mice were heavily infected and died on day 19 and 20. And another 5 mice with severe neurological symptoms were euthanized for animal welfare reasons; In the group of piscidin (0 μg/ml) + PRV, 2 mice were heavily infected and died on day 5. And another 7 mice with severe neurological symptoms were euthanized for animal welfare reasons; In the group of piscidin (10 μg/ml) + PRV, 1 mouse was heavily infected and died on day 19. And another 6 mice with severe neurological symptoms were euthanized for animal welfare reasons; In the group of piscidin (5 μg/ml) + PRV, 3 mice were heavily infected and died on day 19 and 20. And another 7 mice with severe neurological symptoms were euthanized for animal welfare reasons; In the group of piscidin (2.5 μg/ml) + PRV, 1 mice were heavily infected and died on day 20. And another 6 mice with severe neurological symptoms were euthanized respectively for animal welfare reasons; In the group of piscidin (0.5 μg/ml) + PRV, 3 mice were heavily infected and died on day 5 and 7. And another 6 mice with severe neurological symptoms were euthanized for animal welfare reasons; A total of 11 mice from all groups survived and were euthanized by the end of the experiment

In the control group of the *in vivo* assay, mice (*n* = 10) were injected with 50 μl of piscidin (200 μg/ml) by the intra-footpad route. All mice survived and behaved normally. PRV was injected into mice with or without piscidin at various dosages (0.5, 2.5, 5, 10 μg/ml). And the surviving mice were re-challenged with PRV on day 14 (Fig. 6). The mice survival rate was recorded for 28 days. PRV infected mice [Piscidin (0 μg/ml) + PRV] began to show clinical signs around 72 h post infection, characterized by swelling of the inoculated foot and occasional itching. By 144 h post infection, most of the PRV inoculated mice showed constant tremors in inoculated leg and distinctive PRV symptoms of excitation, scratching and biting of the foot. By 168 h post infection, severe neurological symptoms of severe pruritus, ataxia, biting and bleeding of the footpad could be observed. The mice in the piscidin treated groups also showed similar symptoms, but symptoms appeared later and not so severe in piscidin treated mice than those in PRV infected mice. And the mice in the control group [Piscidin (10 μg/ml)] remained asymptomatic, posture normal without signs of neurological symptoms. In the group [Piscidin (0 μg/ml) + PRV], 90% of the mice (*n* = 10) died within 10 days showing typical symptoms of PRV infection. Mice (n = 10) that received piscidin at concentration of above 5 μg/ml were obviously protected against PRV infection (Fig. 6). However, one mouse from the 2.5 μg/ml peptide-treated group and nine mice from the 0.5 μg/ml group died within 10 days. To further characterize the prevention effect of piscidin against brain damage induced by PRV, the brain sections of mice from the control group, PRV-infected, and co-injection group were collected respectively and subjected to pathological examination. The blood stasis was observed in the brain of mice from PRV-infected group (Fig. 6a). The brain of mice from co-injection group showed no specific symptom. After 14 days, the surviving mice were re-challenged with PRV at 5 × 10^3^ TCID_50_. The groups co-injected with piscidin and PRV failed to provide protection with the survival rates dropping to 30% at the end of the experiment and the mice in the 5 μg/ml peptide treated group all died.

## Discussion

We previously reported the proof-of-concept usage of AMPs as the bactericidal agents against pathogenic bacteria isolated from pig herds [23]. The five antimicrobial peptides used in this study have been reported to inhibit the growth of various kinds of viruses including HIV, HSV-1, HSV-2, etc. [12–15, 20–22]. However there has been limited information about the inhibitory activity of these peptides against the swine-origin viruses. Considering this, this study evaluated the effect of these peptides against several porcine viral pathogens. Three peptides (caerin, piscidin, maculatin) exhibited inhibitory activity against PRV, PEDV, TGEV, PRRSV, and rotavirus. Plaque reduction assay showed that the PRV infection could be inhibited in a dose-dependent manner by direct treatment of the peptides.

A previous study indicated that caerin and maculatin could inhibit HIV infection without affecting T-cell viability and that the activity of caerin was more potent than that of maculatin [12]. In our research, we further tested the activity of caerin and maculatin against PRV, PEDV and PRRSV. Compared with maculatin, caerin showed better activity against most of the tested viruses except TGEV (Fig. 1). The fact that caerin has a longer α-helix than maculatin, which enables caerin to disintegrate the membrane more potently, might explain the better activity of caerin. Usually, the bacterial membrane and viral envelope were reported to be the main targets of the AMPs. For example, HIV envelope integrity was directly disrupted by caerin and maculatin [12]. However in this study, TGEV and PRV which both acquired their envelopes from PK-15, exhibited different levels of sensitivity to the tested peptides. In addtion to its envelope, physical characteristics of the virus such as viral size, surface area, and morphology might affect its sensitivity. HNP-1 also demonstrated a 1000-fold difference in activity against different enveloped viruses [16]. It should also be noted that the antiviral activity of the AMPs tested in this study is only observed at concentrations that are already somewhat cytotoxic at 48 h and could be even more so at 72 h or longer. To our surprise, piscidin, caerin, and maculatin could consistently inhibit the growth of RV, a non-enveloped RNA virus. Among these three peptides, piscidin showed the strongest inhibitory activity. It has been reported that piscidin possessed significant antiviral activity to fight against both frog virus 3 and channel catfish virus [30]. The reduction of FV3 infectivity resulting from the application of piscidin is due to its capacity to interact with the essential lipid membrane of FV3 [13]. Based on these results, it could be speculated that there might be other targets for these peptides in addition to the viral envelope, which need further exploration.

Lactoferricin B and indolicidin showed weak or no activity against the five tested viruses compared with caerin, maculatin, and piscidin. Robinson et al reported that the IC_50_ of indolicidin against HIV-1 ranged from 67 μg/ml to 100 μg/ml [31]. The weak activity of indolicidin against the tested viruses in this study was probably due to the low concentration we used (50 μg/ml).

It has been reported that AMPs could inhibit both extracellular and intracellular viruses [32–34]. Our plaque reduction assay result indicated that piscidin, caerin, and maculatin could inhibit PRV by directly interacting with the virus. Addition of the peptides pre- or post-infection could not help inhibit the PRV infection. Vancompernolle et al reported that caerin inhibited HIV infection by disrupting the virion envelope [12]. Chinchar et al also reported that amphibian-origin esculentin-2P (E2P) and ranatuerin-2P (R2P) could inactivate the channel catfish virus (CCV) by directly interacting with the virions [35].

The re-emergence of pseudorabies in China since 2011 has caused a huge economic loss to the pig farms. Three PRV strains, i.e. Ea, 152 and HNXX, were chosen for further evaluation of peptide’s antiviral activity. The peptides inhibited replication of the three PRV strains. This is of great importance in fighting against the mutant viruses resulting from natural selection or antibody-induced events. On the other hand, these peptides had some cytotoxicity on PK-15 cells (Fig. 2).

Piscidin has been reported to have antiviral and antibacterial properties *in vitro* [13, 36]. However, this had not been tested *in vivo*. In this study, the data showed that co-injection of PRV with piscidin at the concentration of above 5 μg/ml could offer protection against PRV infection. Even when the concentration of piscidin decreased to 2.5 μg/ml, the survival rate of mice could reach 90%. Huang et al previously reported that TH1–5, as an AMP isolated from tilapia, could offer 80% protection against JEV infection at 100 μg/ml [37]. Compared with TH1–5, piscidin was more effective even at low concentration. As Huang et al noted, TH1–5 treated mice surviving from the first JEV infection remained alive after second challenge with JEV at day14 [37]. However, our *in vivo* studies exhibited a different result. The possible reason needs further exploration.

## Conclusion

This study indicated that piscidin, maculatin and caerin could inhibit the infection of PRV, PRRSV, PEDV, TGEV and rotavirus. Among the peptides examined in this study, piscidin showed the strongest antiviral activity against PRV both *in vitro* and *in vivo*. And it could block the PRV-induced cell apoptosis as well.

## Data Availability

Data and materials are available upon request by the corresponding author.

## Abbreviations

AMP: Antimicrobial peptide
PRV: pseudorabies virus
TCID_50_: Tissue Culture Infective Dose
